# Experiences of the Summer

**Published:** 1916-10

**Authors:** 


### Experiences of the Summer.

This editorial may prove to be the most valuable one presented but it is not worth reading unless those who read it will contribute their own experience. It seems best to bring up this subject while it is still fresh in the minds of the profession. Whatever replies it elicits, will be assembled, with due credit and published at the beginning of the next summer.

It is stated that rag weed is responsible for 85% of autumnal hay fever, golden rod for practically none but that any plant with an abundance of fine, light, anemophoric pollen may cause the disease. Rose fever seems to be uncommon although many persons imagine they are susceptible to it. Is it analogous to rag weed fever or merely anaphylaxis from volatile oil? Sometimes symptoms resembling mild hay fever arise from dust and some consider dust that includes manure toxic from contained bacteria. Does any kind of hay or grass, by pollen or otherwise, produce hay fever? Why is the summer cold so obstinate? Is it simply a catarrhal inflammation, accepting either the cold or the bacterial theory, or does it partake of the nature of pollen fever? Have you observed cases of asthma excited by feathers, cats, etc., or that illustrate the old idea that two asthmatics or hay fever sufferers might exchange locations and both be relieved? To what extent are bronchial conditions, inflammatory or spasmodic to which a person is subject at any time, confused with hay fever? Have you noted beneficial results from pollen extracts and, if so, to what degree must specific pollens be used?

Have you observed serious cases of ophiophobia or interesting ones of ophiophilia?—or analogous phobias and philias regarding spiders, toads, worms, etc.? We are rather sceptic regarding the existence of a true zoophilia amounting to a perverted psychic condition. For instance, one who really has no special fondness for dogs and cats may hold one on his lap and pet it. A person who is entirely free from ophiophobia might appear a perverted ophiophile to the many persons who have pronounced ophiophobia. Yet we have observed a case of so-called ophiophilia associated with well marked sexual perversion, in a woman.

Reports of bites by poisonous snakes are of course of value, especially if confirming or opposing belief in definite therapeutic measures, as the now almost abandoned faith in alcohol pushed to excess, in permanganate treatment, cauterization, suction of wound, specific serums, etc. There has been some chemic confirmation of the old notion of toxicity of the skin of toads. Reports are desired bearing on this question and as to the toxicity of other animals, especially spiders and insects, and particularly with reference to laryngeal, ocular and other specially dangerous locations of stings. In our camp experience, we have found nothing superior to clean mud as an application to any form of insect bite on the skin.

We are growing somewhat sceptic concerning the commonness of susceptibility to rhus toxicodendron. A surprising number of persons do not even know the plant and the lower animals run through it frequently. It would seem that if the toxicity of this plant is anywhere near so great as is reputed, poisoning would be extremely common. The writer has always been immune and most persons seem to be. We have seen slight urticaria due to contact, relieved simply by washing in lake water at the time and a few other more serious cases—though none of high grade—relieved by lead washes or almost any other soothing lotion. As to the effect of volatile substances or pollen blown by the wind and as to annual recurrences without subsequent direct exposure, we are more than sceptic.

We have seen a few serious cases of sunburn, although serious rather from secondary infection than from the direct results of the dermatitis. It should not be forgotten that, opposed to the great majority who believe in heliotherapy, there are a few who regard the sun's direct rays as always harmful, producing various trophic lesions. We used heliotherapy in a case of intestinal tuberculosis in 1904, the patient being still alive and in fair health, with no present indication of tuberculosis. But even a minority opinion is worth investigating.

There are quite a number of indigenous or introduced poisonous plants. With the exception of rhus toxicodendron and rhus venenata, we believe that none are likely to cause serious danger except to small children eating the berries, or chewing other parts of the plants. Theoretically, the green parts of potato and egg plant and most parts of the other solanaceae, including the deadly night shade, are poisonous. The mints are toxic in large dose and the umbelliferae in less dose. Some persons are even afraid of parsley and parsnips, carrots, etc. We have noted poisoning from tincture of larkspur used to kill lice in the hair, temporary syncope and deafness from a teaspoonful of peppermint oil, and, of course, poisoning from the vegetable abortifacients. Reports of plant poisoning from accidental eating of the green parts, excluding pharmacologic preparations, should be carefully reported.

There is no question but that swimming, which seems to be gaining in popularity, has a hygienic value exceeding its inevitable risk, not to mention its potential value in preserving life in deep water—which is not very great in a storm, or against prolonged immersion in cold water as the ocean, or in accidents in which large numbers of persons are involved. Personally, we have not noticed ill effects from entering the water when heated nor soon after meals. As to the contraindications afforded by menstruation and advanced pregnancy, we have nothing to offer. It has seemed to us that crowded bathing places, even on the shores of large natural bodies of water, present a considerable risk of infection and that even without this factor, prolonged and continued bathing causes susceptibility to ordinary pyogens, so that boils or carbuncles tend to develop or even more serious pyogenic processes ultimately from other predisposing causes. We feel strongly that everyone should leave the water as soon as chilliness becomes a marked subjective symptom and without waiting for blueness of the finger nails and pallor of the face to develop. Yet the numerous instances of persons who violate this rule with apparent impunity must be considered.

We have reiterated warnings against the unsanitary conditions of camps and summer resorts, especially with reference to typhoid for many years. With the present attention given this matter by boards of health and hygienists and the definite recognition given to typhoid as a vacational disease, it is unnecessary to dwell on this subject, except to say that every physician whose sons or patients are contemplating a camping trip, should instruct them in the rudiments of latrine construction, as practiced by armies, for varying periods of encampment. Yet, from the psychologic standpoint, it is worth while to remark on the very general human tendency to burn its sanitary bridges behind it. Even a cat has by instinct the decency to bury its excrement though, when extremely domesticated, like the dog, this instinct may have atrophied into a few symbolic scratches at the dirt. Man, having ascended still farther, uses by choice, a traveled path or a temptingly shady nook which the next comer would use for a resting place. Practically every outing place visited by any large number of persons becomes so foul in the course of a week of dry weather that it is offensive to the sense of smell. Rains wash away the immediate gross contamination into streams and lakes where the after effects may or may not be dangerous, according to the nature of intestinal flora and the use of the water for drinking. Only the winter's rest from such defilement renders it possible for cities to have an outlet into the country for the mass of their population that would not be more dangerous than confinement within the city limits. The same selfish tendency is noted with regard to picnic garbage, waste paper and glass on the beaches. A party of joy riders goes so far as to smash bottle behind their own tires and the average automobilist, delayed by a puncture, leaves the nail in the road for the next man. On the whole, it is possible to argue that the psychology of the general subject exceeds in importance the direct sanitary considerations.

